# Nicotine delivery and relief of craving after consumption of European JUUL e-cigarettes prior and after pod modification

**DOI:** 10.1038/s41598-021-91593-6

**Published:** 2021-06-08

**Authors:** Nadja Mallock, Andrea Rabenstein, Solveig Gernun, Peter Laux, Christoph Hutzler, Susanne Karch, Gabriele Koller, Frank Henkler-Stephani, Maria Kristina Parr, Oliver Pogarell, Andreas Luch, Tobias Rüther

**Affiliations:** 1 Department of Chemical and Product Safety, German Federal Institute for Risk Assessment (BfR), Max-Dohrn-Str. 8-10, 10589 Berlin, Germany; 2 Department of Biology, Chemistry, Pharmacy, Institute of Pharmacy, Freie Universität Berlin, Königin-Luise-Str. 2+4, 14195 Berlin, Germany; 3 Department of Psychiatry and Psychotherapy, University Hospital, Ludwig Maximilian University of Munich (LMU), Nussbaumstrasse 7, 80336 Munich, Germany

**Keywords:** Addiction, Risk factors, Public health, Health policy

## Abstract

The emergence of e-cigarettes on the consumer market led to a tremendous rise in e-cigarette consumption among adolescents in the United States. The success of JUUL and other pod systems was linked to its high nicotine delivery capacity. In compliance with the European Tobacco Product directive, liquid nicotine contents in the European JUUL variants are limited to 20 mg/mL or below. A short time after launching the initial version in Europe, JUUL pods have been modified in terms of the wick material used. This modification has been demonstrated previously to lead to an elevated aerosol generation, consequently, to a larger amount of nicotine per puff generated. The present study was designed to assess whether the mentioned differences between the “initial” and “modified” JUUL versions may cause a significant difference during consumption, and how nicotine delivery compares with tobacco cigarettes. In this single-center three-arm study, nicotine pharmacokinetics and influence on urge to smoke/vape were compared for tobacco cigarettes, the “initial” version of the European JUUL, and the “modified” version of the European JUUL. Participants, 15 active smokers and 17 active e-cigarette users, were instructed to consume their study product according to a pre-directed puffing protocol. Venous blood was sampled for nicotine analysis to cover the acute phase and the first 30 min after starting. Nicotine delivery and the reduction of urge to smoke/vape upon usage of both European JUUL variants were lower in comparison to tobacco cigarettes. This suggests a lower addictive potential. Modification of the pod design did not result in significant differences at the first ten puffs, as confirmed by a vaping machine experiment. Apparently, the limitations by the initially used wick material only come into effect after longer usage time.

## Introduction

Tobacco smoking is a major avoidable health risk, accounting for more than 8 million premature fatalities every year including second hand exposure^1^. The World Health Organization has recognized tobacco consumption as “global epidemic” that must be counteracted by intensified efforts of tobacco control^2^. Smoking cessation is difficult as nicotine is a strong incentive for continued smoking leading to addiction^3^. Diseases induced by cigarette smoking are predominantly linked to hazardous constituents and combustion products, such as the carcinogens benzo[*a*]pyrene, 1,3-butadiene, and formaldehyde^4^. In recent years, a wide range of nicotine delivery devices has entered the market. E-cigarettes aerosolize liquids consisting of propylene glycol and glycerol and optionally containing nicotine and different aroma compounds. Liquids can also contain toxicologically relevant ingredients^5,6^ or impurities as for example tobacco-specific nitrosamines^7,8^. Further, hazardous substances like carbonyl compounds^6^ or flavorant-solvent adducts^9,10^ can be formed during heating. Typically, levels of tobacco related toxicants are strongly reduced in the aerosol as compared with cigarette smoke^11^. Consequently, exclusive use of vaping products facilitates a significantly reduced toxicant exposure in comparison to smoking including dual use of cigarettes and e-cigarettes^12^. Some harm reduction strategies encourage a complete switch to e-cigarettes for smokers who are unable to quit cigarette smoking^13^. Putative health benefits are not anticipated for dual users who consume e-cigarettes and conventional cigarettes in parallel. An increased exposure to tobacco toxicants has even been reported for dual users, as compared with exclusive smokers of conventional cigarettes^12^. To support a complete refrain from combustible cigarettes, nicotine delivery by e-cigarettes needs to be sufficiently high to provide an alternative^14^. Importantly, e-cigarettes with high nicotine delivery are addictive and discussed for non-smokers as a gateway to smoking^15,16^. Whereas early e-cigarettes showed only a limited capacity to reach nicotine blood levels in the range of combustible cigarettes, this has been achieved by newer product generations^17,18^. The US American version of the pod e-cigarette JUUL has a low vaporization power but contains high nicotine concentrations of up to 5% (59 mg/mL) in the liquid^19^. Nicotine delivery in previous studies was comparable to combustible cigarettes in experienced e-cigarette users^20^ but lower in e-cigarette naïve participants^21,22^. This shows that the American version of JUUL has the potential for a high nicotine delivery that is dependent on inhalation and user’s experience with e-cigarettes. The number of adolescent vapers has risen in the recent years, causing a “public health epidemic”, as stated by the US Surgeon General^23^. Signs of nicotine dependence have been reported for adolescent users of pod e-cigarettes^24^. In a recent US study, 13.5% of adolescents and young adults were ever users of the e-cigarette brand JUUL, 6.1% were current users^25^. Reasons for the high acceptance and attractiveness of this e-cigarette brand presumably include curiosity, appealing smell due to the used flavors, convenience of use, initial marketing that was targeting young people, an inconspicuous product design that has led to internet challenges, product use by peers, as well as a high nicotine delivery^26–29^.

JUUL sales in Europe have started end of 2018 with a rather low-key marketing. A comparable impact on nicotine consumption by young people has not been reported for Europe. The big market success as seen in the US failed to repeat in Europe. Furthermore, JUUL labs have announced a withdrawal of JUUL e-cigarettes from some European countries such as Austria, Germany, and Switzerland^30^. The European Tobacco Product Directive (TPD) limits nicotine content in e-liquids to 20 mg/mL^31^. Accordingly, the European version of the product contains only 18 mg/mL nicotine in the liquid, approximately a third of the concentration in the high nicotine US version. Initially, the nicotine level in emissions generated with a vaping machine using a puffing regimen for e-cigarettes (CRM 81) was three times lower when compared to the US version^32^. After a technical modification of the wick material inside the pods that provides liquid to the coil, vapor generation of the EU version increased and nicotine content per puff approximated the US version^32^. A recent human consumption study showed a lower nicotine delivery for the European JUUL version^33^, but did not analyze whether this can be improved by the technical modifications of the different European products. However, data on the nicotine delivery by different versions of JUUL can provide general insight into the capacity and limitations of modern low powered e-cigarettes and is required for their risk assessment. We have assessed parameters such as nicotine pharmacokinetics, urge to smoke, and adverse effects after consumption of JUUL e-cigarettes and conventional cigarettes for experienced e-cigarette users and cigarette smokers using a pre-directed puffing regimen.

## Methods

### Aim and ethics

Aim of the study was to get information about the addictive potential and addiction satisfaction of the European JUUL (“initial” and “modified” versions) compared to a tobacco cigarette. Therefore, we analyzed nicotine delivery of these products, especially in the acute phase, by examining venous blood plasma. The study was approved by the ethics committee of the LMU Munich (Amendment to project number 72-15) and performed in accordance with the principles of the Declaration of Helsinki in the currently valid version. It was registered at the DRKS (DRKS00017432). Informed consent was obtained from all participants before participation in the study.

### Study products and groups

The study was designed as a single-center three-arm study. The products we used were (a) commercial, combustible tobacco cigarettes (Marlboro Red, Philip & Morris), (b) JUUL e-cigarettes with the new technology (JUUL “modified”) with rich tobacco flavor, and (c) JUUL e-cigarettes with the old technology (JUUL “initial”) with rich tobacco flavor. As previously published, the wick used in “modified” JUUL pods consist of a different material than the wick used in the “initial” JUUL pods^32^. Participants received the same instruction for use of both JUUL variants according to the producer manual. Products were purchased in local stores in Berlin and Munich, Germany, and online.

### Participants

This single-center three-arm study included 15 active smokers and 17 active e-cigarette users who were tested with one or both products. 15 sessions were performed for cigarettes, 15 for the modified JUUL, and 11 for the initial JUUL version. This gives a total of 41 experimental sessions. Data from one participant that did not show any increase in nicotine plasma concentration were excluded from analysis. The participants were divided into either the tobacco cigarette group or one of the e-cigarette groups according to the product they normally used. The participants were recruited for participation in the study via advertisement with flyers and the internet. Participants were enrolled in the study after inclusion and exclusion criteria had been checked and participants had provided written informed consent. Inclusion criteria for all volunteers: Age between 18 and 55 years, 12 h of abstinence (e-cigarette and tobacco cigarette consumption), CO levels < 5 ppm (measurement in the expiratory air using a micro-smokerlyzer; Bedfont Scientific Ltd., Anif, Austria) to verify smoking abstinence, and ability to give consent. Special inclusion criteria for electronic cigarette users were e-cigarette use for > 3 months, daily consumption, no daily consumption of conventional tobacco cigarettes for > 3 months. Special inclusion criteria for tobacco cigarette smokers were daily smoking for > 5 years, consumption of > 10 cigarettes/day.

Exclusion criteria for both (electronic cigarette users and tobacco cigarette smokers): Participants under 18 or over 55 years of age, acute psychiatric illness according to ICD-10/DSM IV, other serious psychiatric disorders, acute suicidality, existing pregnancy, breastfeeding, drug, medication, or alcohol abuse at the time of the study, malignant cancer in the past 5 years, serious internal illness, especially cardiovascular diseases, such as manifest arterial hypertension, severe heart disease (DCM, history of heart attack), pacemaker implantation, respiratory failure, severe active infectious disease. E-cigarette users were invited to participate in both JUUL study arms. Thus, 9 participants used both JUUL variants, 6 participants used only the “modified” JUUL, and 2 participants used only the “initial” JUUL.

### Study design and questionnaires

Clinical data were collected during January and September 2020. Two appointments were scheduled for the test subjects to participate in the study. The first appointment was considered a screening, whereas the actual measurement took place at the second appointment. Usual smoking or e-cigarette consumption behavior was enquired with standardized and specially designed questionnaires.

An initial questionnaire at the screening appointment served on the one hand to assess sociodemographic data such as sex, age, weight and known pre-existing illnesses and on the other hand to assess smoking and e-cigarette consumption behavior. For example, the frequency of smoking or vaping and preferred manufacturers were assessed. At the screening appointment, physical dependence on nicotine was assessed with the Fagerström Test of Nicotine Dependence (FTND) according to Heatherton et al*.*^34^. No validated version of the FTND exists for e-cigarette consumption. Thus, instead of the FTND, an adapted but unvalidated questionnaire for e-cigarettes was used for participants in the JUUL study arms. The Questionnaire on Smoking Urges (QSU-G) in the German version by Müller et al*.* was used to assess craving^35^. QSU-G was assessed before and immediately after the vaping/smoking sessions. Further details on QSU-G and its evaluation are given in the Supplementary Information. Immediately after vaping, participants rated negative effects (side effects) of the e-cigarette on a visual analog scale (VAS) ranging from 0 (no effect) to 10 (strongest effect). The VAS used in this study was based on the one used in previous studies on e-cigarettes^36,37^ and enquired urge to vomit, nausea, perspiration, headaches, palpitations, cold hands or feet, salivation, dizziness, irritation of the throat or mouth, and lightheadedness.

E-cigarettes were weighed (MC 1, Sartorius, Göttingen, Germany) before and after the measurement to determine the liquid consumption. Nicotine doses were calculated by considering liquid density (1.16 g/cm^3^) and the measured nicotine concentration in the respective e-liquid (17.69 mg/mL for “modified” JUUL and 17.20 mg/mL for “initial” JUUL)^32^.

### Puff topography

**Figure 1:**
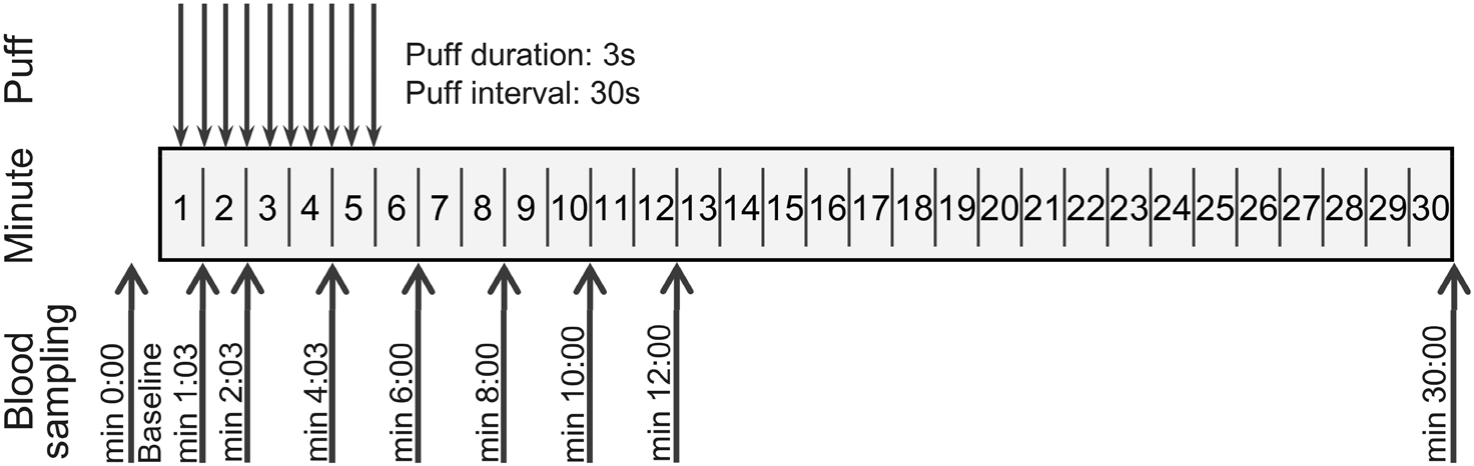
Study design.

The consumption sessions were carried out according to Fig. 1. A puff duration of 3 s was selected according to a recent study on pod e-cigarettes^38^ and to a well-established regime for machine puffing of e-cigarettes^39^. It was ensured that the puffs were 3 s long and that the blood samples were taken after the completed puff. In total, 10 puffs were taken, heart rate and blood pressure were measured 4 times, and 9 blood samples were taken. A metronome was used to standardize the duration of the inhalations by providing an acoustic signal at the beginning and end of inhalation. The study investigator instructed all study participants to inhale in exactly the same way at each inhalation and study visit. Participants were instructed to inhale the product aerosols into their lungs.

### Blood sampling

A peripheral venous cannula was inserted to allow blood samples to be taken at short intervals. To determine nicotine, cotinine, and *trans*-3′-hydroxycotinine (hydroxycotinine) levels, a total of nine blood samples with 7.5 mL each were taken at various time points before, during and after smoking/vaping as presented in Fig. 1. They were carried out in accordance with the generally applicable hygienic standards using Safety Multifly cannulas and S-Monovettes. Blood was placed on ice immediately after sampling until centrifugation (10 min, 1500*g*, 4 °C). Internal standard mix (10 µL of 500 ng/mL nicotine-d_3_, cotinine-d_3_, hydroxycotinine-d_3_ in acetonitrile) was added to plasma samples (990 µL).

### Analysis of nicotine, cotinine, and hydroxycotinine plasma concentrations

Nicotine and its main metabolites cotinine and hydroxycotinine were analyzed using LC–MS/MS with a validated method as published previously^40^. Blood samples were centrifuged at 1500*g* and 4 °C for 10 min to obtain plasma. 990 µL plasma was spiked with 10 µL internal standard mix (500 ng/mL (±)-nicotine-(methyl-d3), (±)-cotinine-(methyl-d3), and *trans*-3′-hydroxycotinine-d_3_ in acetonitrile) at LMU in Munich and shipped on dry ice to the BfR in Berlin. For protein precipitation, 100 µL ice-cold methanol were added to 50 µL plasma sample and then centrifuged at 4 °C and 14,000*g* for 15 min (Centrifuge 5427 R, Eppendorf, Wesseling-Berzdorf, Germany). Supernatant was diluted 1:1 with mobile phase A (see below) prior to injection of 25 µL into the LC–MS/MS system (LC: Prominence series from Shimadzu, Kyoto, Japan; MS/MS-System: API4000QTrap, AB Sciex, Framingham, MA, USA). For separation, a Luna Phenyl-Hexyl Column (150 mm length × 4.60 mm I.D., 3 µm particle size, 100 Å pore size; Phenomenex, Torrance, CA, USA) with an according guard column (Phenomenex, Torrance, CA, USA) was used at 45 °C at a flow rate of 1 mL/min. As eluent A, 5 mM ammonium acetate in water, pH adjusted to 4.50 ± 0.02 with formic acid was used and for eluent B methanol. The gradient was as followed: Started at 10% B, increase for 1 min to 30% B, hold for 1 min, increase for 2 min to 95% B, hold for 2 min, decrease for 0.2 min to 10% for 0.2 min, and a hold for 2.8 min. ESI–MS/MS parameters are provided in the Supplementary Information. Nicotine, cotinine, and hydroxycotinine were quantified using a matrix matched calibration.

### Machine vaping

To mimic vaping, 10 puffs were taken from JUUL devices equipped with 5 freshly opened “modified” JUUL pods or 4 freshly opened “initial” JUUL pods using a linear vaping machine for e-cigarettes (LM4E with PM1 piston pump, Borgwaldt, Hamburg, Germany). By the time the machine vaping part was performed, “initial” pods were already taken off the market. Thus, only 4 pods could be analyzed. CORESTA Reference Method 81 was applied: puff duration 3 s, puff frequency 30 s, puff volume 55 mL, rectangular puff profile^39^. Emissions were collected on glass fiber filter pads (Borgwaldt, Hamburg, Germany) that were exchanged after 2 puffs during the 30 s inter-puff interval. Total particulate matter (TPM), the weight gain in the filter, was determined by weighing (LE225-0CE, Sartorius, Göttingen, Germany). Nicotine dose was calculated by dividing by liquid density (1.16 g/cm^3^) and multiplication with the nicotine concentration in e-liquid (17.20 mg/mL for the initial and 17.69 mg/mL for the modified version) as previously determined^32^. As previously shown, the nicotine content in the aerosol can be calculated on the basis of the liquid consumption leading to similar results as determined with GC-FID^32^. This is in line with other studies^41^. Further, we have previously shown that weight loss of the liquid (liquid consumption) and weight gain of the glass fiber filter (TPM) are comparable^32^, also in line with the literature^41^.

### Pharmacokinetic (PK) parameters and statistical analysis

Statistical analysis was performed with Statistical Software Program System (SPSS) version 21.0. Data derived from QSU-G were analyzed with the t-test for paired samples. Areas under the plasma concentration–time curve (AUC) were calculated after baseline correction (subtraction of C_t0_) applying the linear trapezoid rule. C_max_ and t_max_ were the highest nicotine concentrations per individual plasma curve and the according time points. Participants were asked to stay nicotine abstinent overnight. However, nicotine PK parameters were reported with and without baseline correction (subtraction of C_t0_) to control potential high nicotine baseline effects, possibly due to intensive smoking on the previous evening. For statistical analysis of C_max_ and AUC, geometric mean and CV were used, and a two-sided unpaired t-test was used with lognormal values to test for statistical significance. For mean plasma curves, arithmetic means of baseline corrected concentrations at each time point were calculated. Nicotine metabolic ratio (NMR) was calculated as a surrogate for CYP 2A6 metabolic activity^42,43^ by dividing hydroxycotinine plasma concentration by cotinine plasma concentration at t_0_ when metabolites were detected. For NMR and other participant characteristics, median and IQR were calculated. Arithmetic mean and standard deviation were used for liquid consumption, nicotine dose, and TPM.

## Results

### Participants

Participant characteristics such as age, sex, FTND score, nicotine metabolic ratio, and product use characteristics are summarized in Table 1. The level of physical nicotine dependence measured by the FTND score ranged from low to very severe in all three groups. In the JUUL (modified) group the level of physical dependence was low in n = 7/15, moderate in n = 2/15, severe in n = 2/15, and very severe in n = 4/15. The JUUL (initial) group showed a low level of physical dependence in n = 4/10, a moderate level in n = 1/10, and a severe level in n = 3/10 and very severe in n = 2/10. One participant in the JUUL (initial) arm did not complete the questionnaire. In the tobacco group, the level of physical dependence was low in n = 11/14, moderate in n = 2/14 and very severe in n = 1/14. The parameters “days smoked/EC used in the past 30 days”, “Pods (0.7 mL) used per day”, and NMR did not differ among groups. One participant in the tobacco cigarette group seemed to have not inhaled during smoking as the plasma nicotine level did not rise higher than 0.1 ng/mL (see Supplementary Information). This participant was excluded from further analysis and calculations of the below presented results. Two participants in the modified JUUL group had to be excluded for PK data analysis because relevant blood sampling time points were missing due to clogging of the cannula and the participants could not be recruited for a repetition.

**Table 1:** Participant characteristics.

Age, median (IQR)	28 (25–33)
Sex, female, n (%)	13 (41.9)
Sex, male, n (%)	18 (58.1)
**Tobacco cigarette group, median (IQR), n = 14**	
Fagerstrom test for nicotine dependence (FTND) before cigarette use	1 (0–2.5)
Cigarettes smoked per day when joining the study	8 (5–11)
Cigarette smokers: days smoked in the past 30 days	28 (25–30)
Nicotine metabolic ratio	0.47 (0.29–0.62)
**JUUL (modified) group, median (IQR), n = 15**	
Fagerstrom test for nicotine dependence (FTND) before JUUL use	3 (2–7)
Pods (0.7 mL) used per day when joining the study	0.9 (0.3–1.3)
Nicotine metabolic ratio	0.39 (0.27–0.49)
**JUUL (initial) group, median (IQR), n = 11**	
Fagerstrom test for nicotine dependence (FTND) before JUUL use	4 (1.5–5.75)
Pods (0.7 mL) used per day when joining the study	1 (0.5–1.3)
Nicotine metabolic ratio	0.43 (0.26–0.51)
**All JUUL users, median (IQR)**	
JUUL (both) users: days EC used in the past 30 days	30 (30–30)

### Nicotine delivery from different study products

**Figure 2:**
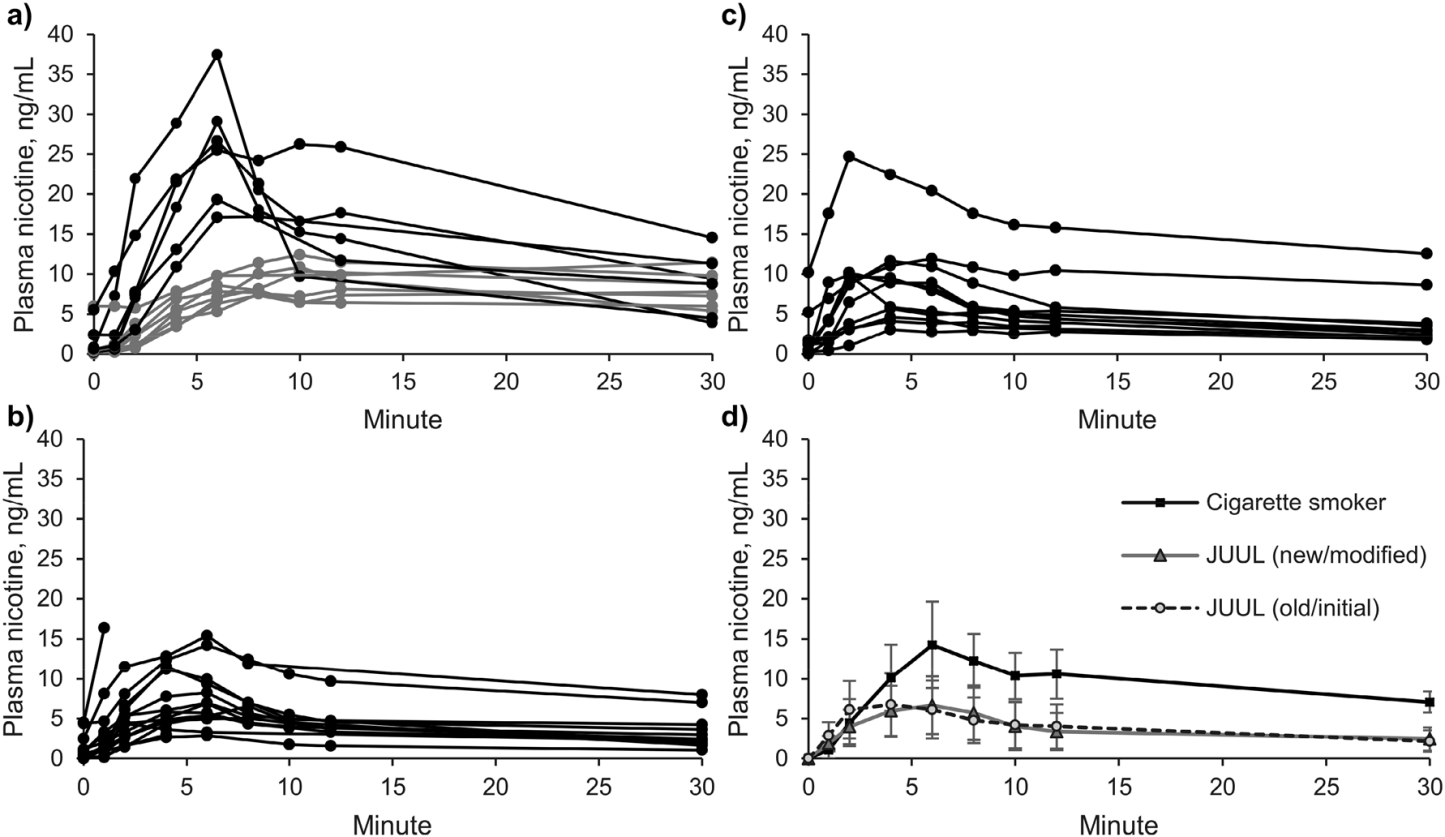
Individual nicotine plasma curves without baseline correction (C_t0_ subtraction) after use of (**a**) tobacco cigarettes (n = 14), (**b**) JUUL (modified) e-cigarettes (n = 15), and (**c**) JUUL (initial) e-cigarettes (n = 11). (**d**) Arithmetic means and 95% confidence interval of the plasma curves from three groups.

Plasma nicotine curves from all participants are displayed in Fig. 2 as spaghetti plots without baseline correction (subtraction of C_t0_) (Fig. 2a–c), and as arithmetic means with baseline correction and 95% confidence interval (Fig. 2d). Nicotine levels for each participant and time point in addition to individual liquid consumptions can be found in the Supplementary Information. For cigarette smokers, two different plasma curve shapes were apparent: 6 cigarette smokers had C_max_ values of above 15 ng/mL with t_max_ values of approximately 6 min (Fig. 2a, black lines), 8 cigarettes smokers had C_max_ values of below 15 ng/mL with t_max_ values of about 10 min (Fig. 2a, grey lines). For some further analysis and discussion, smokers were divided according to these two plasma curve types into “low C_max_” smokers (C_max_ < 15 ng/mL) and “high C_max_” smokers (C_max_ > 15 ng/mL). Median FTND score of “high C_max_” smokers was with 1.5 (IQR 0–4.75) slightly higher than of “low C_max_” smokers” with 0.5 (IQR 0–1). Further, NMR was with 0.59 (IQR 0.46–0.66) slightly higher for “low C_max_” smokers” than for “high C_max_” smokers with 0.34 (IQR 0.25–0.49) but not statistically significant (p = 0.1).

Relevant PK parameters such as C_max_ with and without baseline (C_t0_) correction, AUC, t_max_, liquid consumption, and calculated nicotine doses are shown in Table 2. Differences between both JUUL variants are small and non-significant for all PK parameters. Further, liquid consumption was the same for both JUUL variants. C_max_ and AUC after JUUL consumption were approximately 40–50% smaller than after tobacco smoking (p < 0.005). Plasma curves for metabolites cotinine and hydroxycotinine are shown in the Online Supplementary Information.

**Table 2:** Relevant PK parameters for the different study products and a comparison between the “modified” JUUL version and tobacco cigarettes.

	Tobacco cigarette n = 14	JUUL (modified) n = 13	JUUL (initial) n = 11	JUUL (modified) vs. tobacco cigarette
C_max_ (ng/mL) without C_t0_ correction	14.4 (73%)	7.2 (74%)	8.1 (81%)	50% (p = 0.002)
C_max_ (ng/mL) with C_t0_ correction	13.1 (77%)	6.3 (69%)	6.5 (79%)	48% (p = 0.001)
AUC_0–30 min_ (ng/mL min) with C_t0_ correction	257.0 (49%)	103.3 (63%)	110.9 (49%)	40% (p = 0.00005)
t_max_ (min)	8 (6–30)	6 (2–8)	4 (2–6)	
Liquid consumption (mg)	N/A	31.9 ± 8.3	30.6 ± 10.9	
Nicotine dose (mg)	N/A	0.49 ± 0.13	0.47 ± 0.17	

C_max_ (with and without C_t0_ correction) and AUC: Geometric mean and coefficient of variance (CV%); t_max_: Median and range; Liquid consumption and nicotine dose: Arithmetic mean and standard deviations (SD); p-values obtained with unpaired, two-sided t-test with logarithmic values.

### Craving

**Figure 3:**
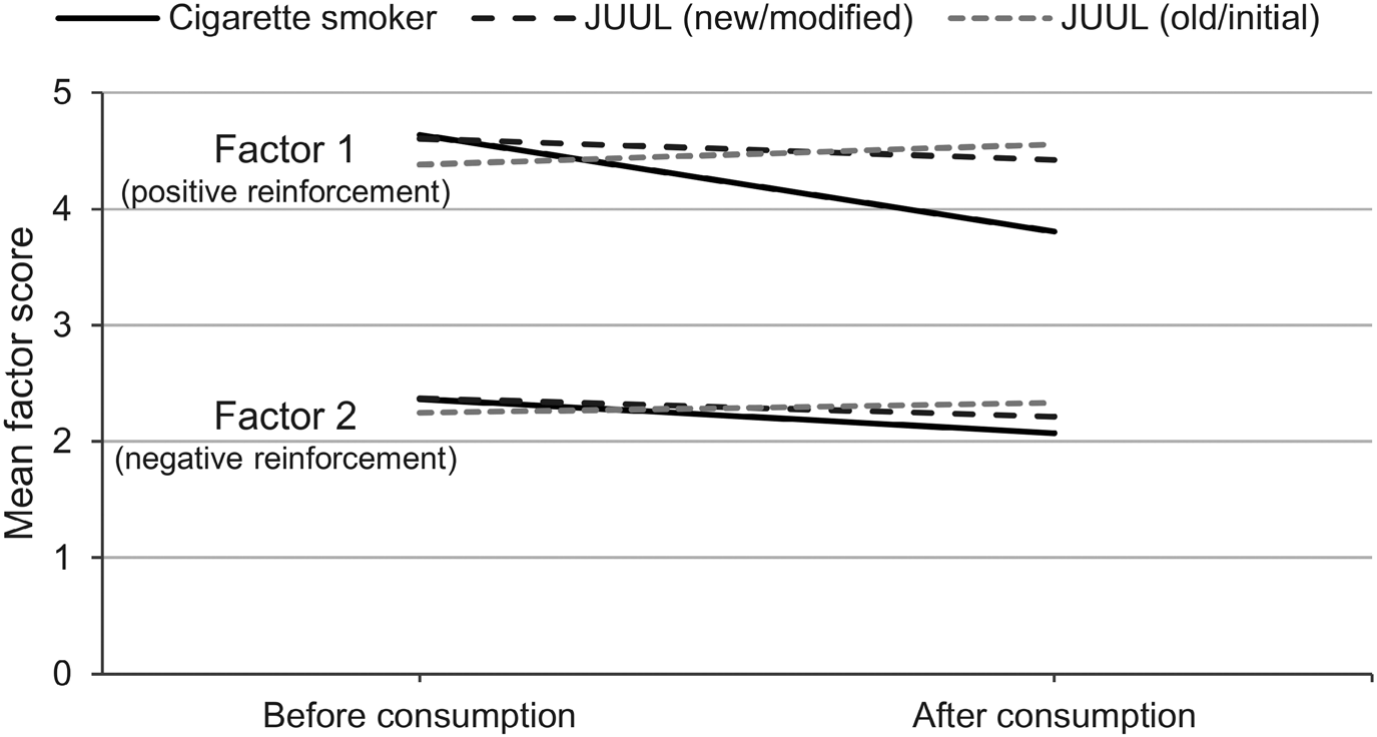
Mean scores of urge to smoke or urge to vape before and after consumption for all three product groups [combustible cigarette (n = 14), “modified” JUUL (n = 14), “initial” JUUL (n = 10)] divided into factor 1 (positive reinforcement) and factor 2 (negative reinforcement).

Craving factors determined with the QSU-G were divided into factor 1 for positive reinforcement and factor 2 for negative reinforcement. Two participants did not return their questionnaires. Results are shown in Fig. 3. Mean of factor 1 (positive reinforcement) decreased after tobacco cigarette smoking by 0.83, decreased after “modified” JUUL use by 0.18, and increased by 0.17 after “initial” JUUL use. The changes in factor 1 were only significant in the tobacco cigarette group (p = 0.006).

Factor 2 (negative reinforcement) decreased in the tobacco cigarette smoke by 0.29 and in the “modified” JUUL group by 0.15. Factor 2 increased slightly in the “initial” JUUL group by 0.08. “High C_max_” smokers showed a decrease of 1.62 in factor 1 and 0.77 in factor 2. Decrease in factor 1 in “high C_max_” smokers was significant (p = 0.001). Factor 1 decreased in “low C_max_” smokers by 0.23 and factor 2 increased slightly by 0.06. Survey was not fully completed by 1 participant in the “modified” JUUL arm and from 2 participants in the “initial” JUUL arm.

### Side effects

**Figure 4:**
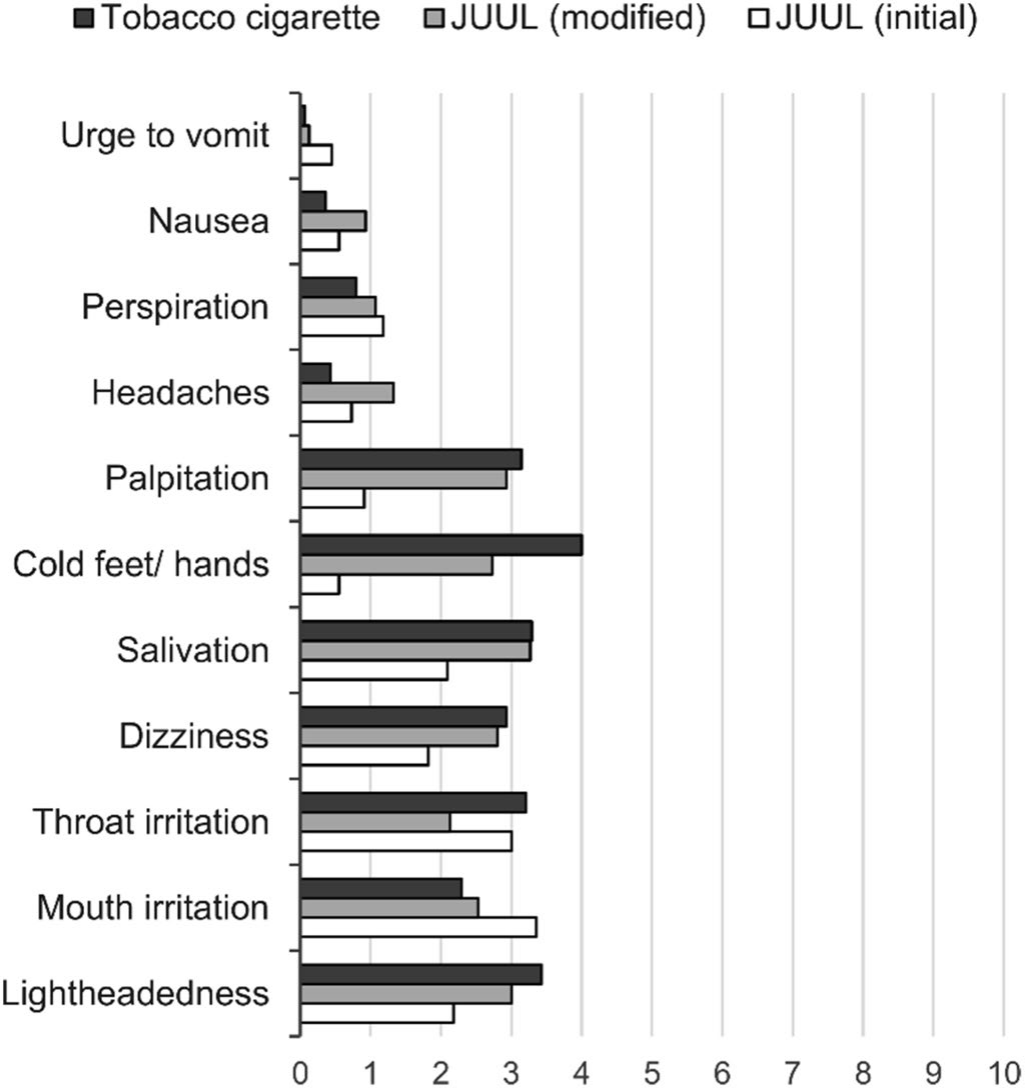
Reported side effects after use of tobacco cigarettes (n = 14), modified JUUL (n = 15), and initial JUUL (n = 11) version on a visual analog scale (VAS) ranging from 0 (no effect) to 10 (strong effect).

Negative side effects were enquired at the end of smoking and vaping sessions. Visual analog scale (VAS) scores are displayed in Fig. 4. VAS scale ranges from 0 (no effect) to 10 (strong effect). Most side effects occurred in cigarette consumption, where the mean overall VAS score was 2.1. The tobacco cigarette group also achieved the highest values for most individual items on the VAS score.

### Acute phase

**Figure 5:**
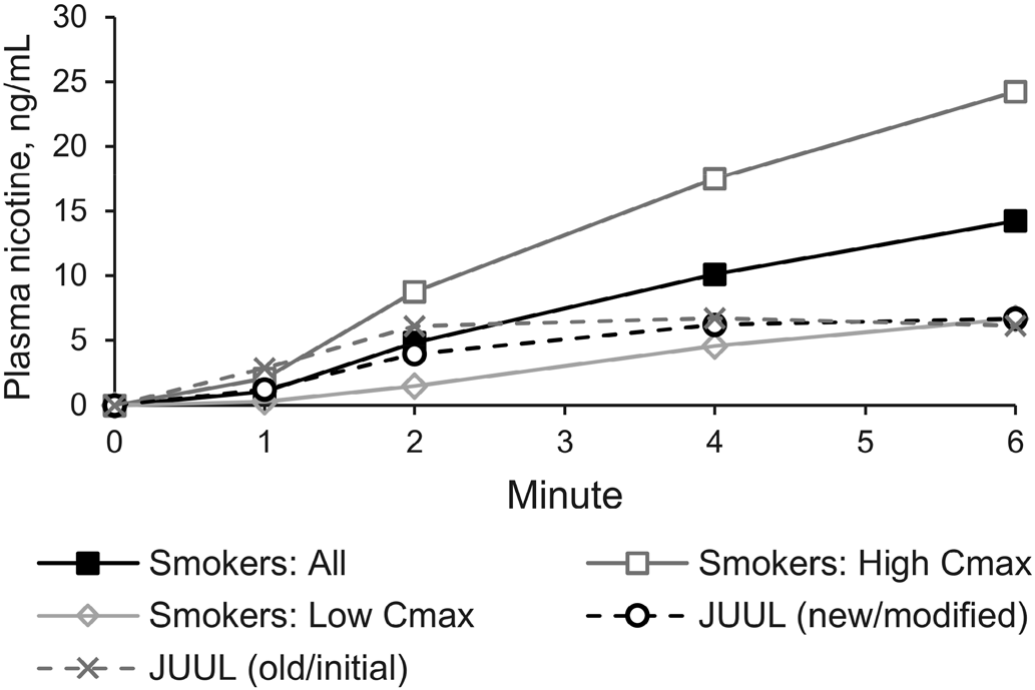
Plasma nicotine concentration in the acute phase. Arithmetic means of nicotine concentrations of the three study groups (tobacco cigarette smokers, JUUL (modified) users, and JUUL (initial) users) and of the smoker subgroups (“high C_max_”, C_max_ > 15 ng/mL; “low C_max_”, C_max_ < 15 ng/mL).

For better comparison of nicotine kinetics in the acute phase, arithmetic means of baseline corrected plasma nicotine concentrations are displayed in Fig. 5 for all three study groups (smokers and users of both JUUL variants) and additionally for the two smoker subgroups (“low C_max_” and “high C_max_” smokers). While the means of nicotine plasma curves from cigarette smokers, especially from all smokers and from “high C_max_” smokers, were rising for at least 6 min, mean nicotine plasma curves from JUUL users flattened after 2–4 min.

### JUUL use simulation with a vaping machine

**Figure 6:**
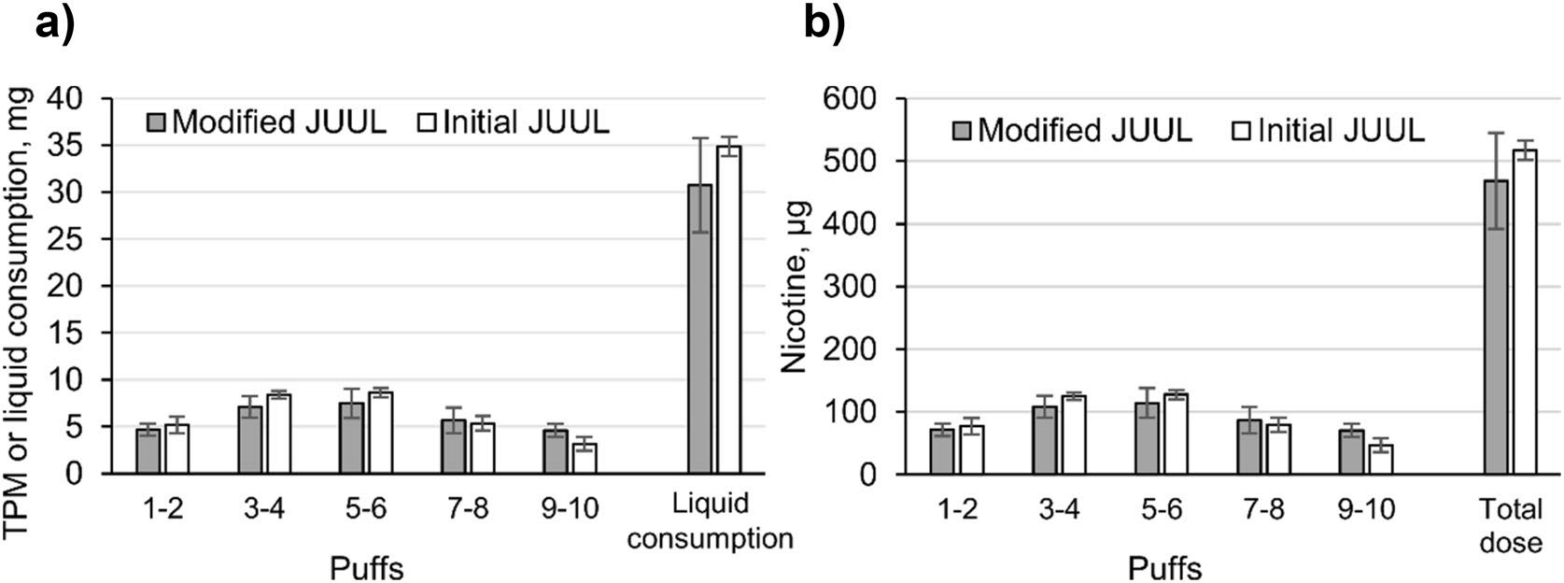
Machine generated vapor per two puffs expressed as (**a**) mean total particulate matter (TPM) with standard deviations for modified (n = 5) and initial (n = 4) JUUL version. Liquid consumption has been calculated by weighing pods before and after use. (**b**) Nicotine dose has been calculated by multiplying TPM with liquid nicotine concentration. Total nicotine dose was calculated using the liquid consumption.

In contrast to combustible cigarettes, both versions of JUUL did not facilitate a continuous rise of nicotine plasma levels during vaping. To investigate whether this was linked to a decrease in vapor generation, a machine vaping experiment was performed. Using a standard puffing protocol (55 mL puff volume, 3 s puff duration, 30 s puff interval, rectangular puff profile), vapor generation in the first 5 min of both JUUL versions was monitored. Figure 6 shows TPM (the vapor that was collected on a filter pad) and the calculated nicotine delivery per 2 puffs for different pods. E-cigarettes were weighed before and after the procedure. The mean total liquid consumption was 30.73 ± 5.03 mg with a calculated nicotine delivery of 468.60 ± 76.65 µg for the modified version and 34.88 ± 1.04 mg consumed liquid and 517.19 ± 15.43 µg nicotine for the initial version. Divided by the puff number, average nicotine doses per puff were 47 µg and 52 µg for the modified and the initial version, respectively.

## Discussion

Our study was aimed to compare performance of the two European versions of the pod e-cigarette JUUL with tobacco cigarettes under defined product using conditions. This means that puff number, interval, and duration has been standardized for all products. Only the puff volume could not be standardized. Adjustment of all parameters is necessary to limit the impact of intraindividual differences when directly comparing the performance of products. Our study was designed to monitor the rise of nicotine blood concentration during the acute phase rather than to reflect real-life product use and nicotine exposure^17^. Data obtained with the pre-directed puffing protocol demonstrate that nicotine delivery from the European version of JUUL e-cigarettes is significantly lower in comparison to tobacco cigarettes: Maximum plasma nicotine concentrations and AUC_0–30 min_ from JUUL users were significantly lower (40–50%) and nicotine curves flattened earlier in the acute phase. The acute phase has been defined in a previous publication as the first 5 min during consumption^17^ and is of special relevance when evaluating addictiveness of different products. Increases of nicotine concentrations in the brain are fastest upon inhalation. Consequently, nicotine inhalation leads to a rush of nicotine in the brain during the initial smoking phase^3^.

These findings are in agreement with the recently published study led by Phillips-Waller et al*.* who compared EU JUUL (20 mg/mL), US JUUL, and tobacco cigarettes^33^. As the initial JUUL version was declared to contain 20 mg/mL while the modified version was labelled with 18 mg/mL^32^, it is assumed that Phillips-Waller et al*.* have used the initial version in their study. They have demonstrated that the US American version with 59 mg/mL delivers comparable amounts of nicotine as tobacco cigarettes in experienced e-cigarette users^33^. The authors have reported a slightly lower nicotine delivery after EU JUUL consumption compared with our results^33^. A recent study by the manufacturer came to lower nicotine deliveries by the European JUUL versions in a study with 5-min ad libitum use sessions and pre-directed use sessions that were similar to the ones in this study^44^. Results from different studies on the same product can differ depending on the study design, especially in terms of user experiences. Inexperienced vapers using the high nicotine US JUUL version did not reach the nicotine delivery level of tobacco cigarettes^21,22^.

For comparison of JUUL with other e-cigarette types, some key features like convenience of use are of relevance. For example, ease of product use was the main reason for the JUUL use continuation in college students^27^. Disposable JUUL pods are sold prefilled with e-liquid and with the heating element included. Modern pod systems with high nicotine contents have the potential to deliver comparable nicotine levels as combustible cigarettes. This marks an important distinction to earlier generation disposable e-cigarettes, so-called cigalikes that delivered nicotine less efficiently in a study using a similar puffing protocol^17^. Nicotine delivery of JUUL e-cigarettes was higher compared to cigalikes (C_max_ = 5.5 ng/mL) and was almost comparable to tank e-cigarettes (C_max_ = 9.3 ng/mL) that are more complex in handling^17^.

Another main question of this study was to follow up whether the modification of JUUL pods by the manufacturer led to an increase of nicotine delivery during consumption. The initially sold European JUUL version had similar design features as the US version except for the approximately threefold lower nicotine contents. In a previous study, it was shown that the wick material in the heating element was exchanged in a modified product version that was launched in summer 2019^32^. The new wick material supposedly has a better capability to supply the heating coil with fresh liquid, resulting in a more stable and a threefold higher vapor generation compared with the initially used wick material^32^. In our study, we have raised the question whether this technical modification translates into an increase of nicotine plasma levels, consequently circumventing the nicotine limits set by the TPD. We have compared nicotine delivery during use of both European JUUL versions. Surprisingly, nicotine delivery and liquid consumption were the same for both variants. In the meantime, influence of wick material has been investigated in a clinical study by the manufacturer resulting in similar findings^44^. To follow up this observation, we simulated the pre-directed puffing protocol (10 puffs, 3 s puff duration, 30 s puff frequency) with a vaping machine, applying the CORESTA Recommended Method 81 (55 mL puff volume and rectangular puffing profile)^39^. Both EU JUUL variants were tested under the same conditions as in the clinical part. Ten puffs were drawn from each pod that was freshly opened. Liquid consumption, generated vapor, and calculated nicotine delivery did not differ between the EU JUUL variants. The overall consumed liquid in the machine vaping experiments was comparable to the mean liquid consumption of both EU variants in the clinical study. According to a mathematical model proposed by Talih et al*.*, puff volume does not influence the mass of generated vapor from an e-cigarette, while puff duration is an important parameter that usually equals the heating time of the coil^41^. In our study, participants were instructed to take puffs of 3 s and were guided by an acoustic signal. Consequently, puff durations in the clinical and the vaping machine part were the same. This explains the good predictability of liquid consumption in the clinical part by the vaping machine experiment. However, this raises questions about the difference in performance that has been detected in the previous study^32^. Amounts of nicotine per puff were previously determined as 61 µg in the emissions of the modified version and 23 µg in the emissions of the initial version. In the previous study, a total of 160 puffs were drawn from each pod in sets of 20 puffs^32^. Average nicotine doses per puff calculated for the herein presented vaping machine experiment were 47 µg for the modified and 52 µg for the initial version. This indicates that performance of both variants at the first ten puffs is close to the performance of the modified version over 160 puffs (61 µg nicotine per puff). In conclusion, we postulate that the wicks are initially saturated with liquid. The disadvantage of the limited liquid supply by the initial wick material only becomes apparent after a larger number of puffs are taken. Then, liquid supply becomes a limiting factor. Vapor generation with the modified wick has been more stable over time^32^, indicating that this disadvantage has been compensated. This could explain that both versions have led to the same nicotine plasma curves after consumption following the pre-directed protocol. Possibly, if more puffs were taken in the clinical part of this study, nicotine delivery by the initial version might even decrease when the liquid supply becomes slower. Nicotine delivery of the modified version is expected to increase only slightly after more puffs are taken. Calculated nicotine delivery per puff when only ten puffs are used was with 47 µg per puff 77% of the nicotine delivery per puff assessed over 160 puffs (61 µg per puff), and thus already close to maximum. This improved version still cannot mimic the nicotine delivery of tobacco cigarettes in contrast to the US version that uses the initial wick material but has a threefold higher nicotine content in the liquid^32^. Interestingly, influence of wick material on puff generation is usually a neglected factor^41^ as it is rather uncommon that the supply to the coil by the wick becomes rate limiting. However, the present case demonstrates that predictability of nicotine delivery can be hampered by unexpected design features that can be of advantage or disadvantage for vapor generation. This should be kept in mind for future investigations on nicotine delivery by devices with uncommon design features.

Besides the nicotine delivery, the urge to smoke and to vape was scored, and side effects were assessed. Assessment of craving was divided into positive and negative reinforcement factors^35^. Positive reinforcement describes the intention to smoke and anticipation of positive effects from smoking. Negative reinforcement indicates the craving for smoking and anticipation of relief from negative effects of nicotine withdrawal^35^. Positive reinforcement factors have been reduced for smokers but not for JUUL users. This agrees with the absence of a notable nicotine peak in the plasma curves derived from JUUL users. Decrease in factors for negative reinforcement was overall low and comparable between cigarette smokers and JUUL users. This low decrease could be linked to the overall low physical dependence of the participants according to FTND scores (see Table 1). Negative side effects were overall low and did not markedly differ among groups. Of special interest were effects such as mouth and throat irritation. Nicotine salt formulations are actually applied to alleviate the irritative effects of high nicotine contents in e-cigarette liquids^45,46^. In the present study, no notable differences were detected between groups in terms of irritative effects.

Further, it should be noted that two different plasma curve shapes were visible among cigarette smokers although the same cigarette brand was used by all participants. Smokers were divided into two subgroups solely based on visible differences in their plasma curve shapes. “High C_max_” smokers showed a plasma curve that is known from smokers with a high rise of blood nicotine levels in the acute phase. C_max_ in all these curves was above 15 ng/mL. “Low C_max_” smokers revealed a more plane curve and a C_max_ of below 15 ng/mL. These participants did not take advantage of the cigarette’s full potential. This could have been influenced by the pre-defined puffing regimen that might be too different from their normal smoking behavior or could have been linked to the low physical dependence score that smokers, especially “low C_max_” smokers, had in the FTND. Further, NMR was higher in “low C_max_” smokers. Higher NMR means faster nicotine metabolism^42,43^. However, these differences were not statistically significant. Plasma curves derived from JUUL were comparable to those of “low C_max_” smokers.

Taken together, the presented results suggest that European JUUL has a lower nicotine delivery in total and in the acute phase in comparison to tobacco cigarettes and the US version (59 mg/mL nicotine) in experienced users despite the product modification. This is in line with the recently published results^33,44^. According to these data, it becomes likely that abuse liability and addictiveness of the European version is lower. This might be one reason why high acceptance noticed in young non-smokers in the US did not become apparent in Europe.

### Limitations and outlook

During 59 from 405 blood samplings, the cannula clogged that was used to draw blood. If possible, a new cannula was placed but some time points were missed. When the blood sampling at expected t_max_ was missing, the participants were contacted for a revisit. Four participants responded and were reinvited. Further, differences in plasma curves between groups of participants could have been influenced by factors such as physical nicotine dependence as measured with FTND and self-titration. Results of studies like this one are highly dependent on the recruitment of participants with different target nicotine blood levels. Although the number of participants was common for this type of study, larger numbers of participants would have helped to avoid the impact of interindividual differences in dependence. A cross-over design would have been beneficial for statistical analysis but would rely on dual users as participants should be experienced with their study product. Further, the study design enforced a vaping pattern that could differ from individual preferences of users. The aim of this study was to compare nicotine delivery by different products versions of JUUL under defined conditions. It was further discussed that JUUL pods have an inconsistent performance depending on puff number. Thus, a long-time ad libitum consumption study could therefore give further insights to the product’s potential nicotine delivery characteristics.

## Supplementary Information

Supplementary Information 1.

Supplementary Information 2.

## Data Availability

All data generated or analyzed during this study are included in this published article (and its Supplementary Information files).
