# Supplementary material for: Nicotine delivery and relief of craving after consumption of European JUUL e-cigarettes prior and after pod modification
Source: Sci Rep. 2021 Jun 8;11:12078. doi: 10.1038/s41598-021-91593-6 (PMC8187405; doi:10.1038/s41598-021-91593-6)
Supplement: Supplementary file 2 — Supplementary Information 2. [file 41598_2021_91593_MOESM2_ESM.docx]

Online Supplementary Material

Nicotine delivery and relief of craving after consumption of European JUUL e-cigarettes prior and after product modification

Nadja Mallock^1,2#^ & Andrea Rabenstein^3#^, Solveig Gernun^3^, Peter Laux^1^, Christoph Hutzler^1^, Susanne Karch³, Gabriele Koller³, Frank Henkler-Stephani^1^, Maria Kristina Parr², Oliver Pogarell³, Andreas Luch^1,2^ & Tobias Rüther^3^

^1^German Federal Institute for Risk Assessment (BfR), Department of Chemical and Product Safety, Berlin, Germany

^2^Institute of Pharmacy, Department of Biology, Chemistry, Pharmacy, Freie Universität Berlin, Berlin, Germany

^3^Department of Psychiatry, Ludwig-Maximilians-Universität (LMU), Munich, Germany

# Authors contributed equally

*corresponding author: Nadja.Mallock@bfr.bund.de

**Supplementary information on methodology**

**Questionnaire on Smoking Urges (QSU-G)**

The Questionnaire on Smoking Urges (QSU) consists of 32 items which are rated on a scale from 1-7 (totally disagree to totally agree). The German version of the QSU (QSU-G) was published in 2001 after a validation study in Germany and has since become an established clinical instrument to evaluate smoking behavior^1^. Participants completed the QSU-G before and after the study visits to determine their craving for smoking. The QSU-G assesses two factor-specific dimensions of subjective craving for smoking on a seven-level rating scale. Factor 1 describes the intention to smoke and anticipation of positive effects from smoking (positive reinforcement, positive reinforcement). Factor 2 indicates the craving for smoking and anticipation of relief from negative effects of nicotine withdrawal (negative reinforcement, negative reinforcement).

For data analysis, test items were grouped into both factors. While the items 4,5,6,9,11,17,21,25,27,28 and 32 represent factor 1 (positive reinforcement), the items 2,3,7,13,18,19,24,29,30 and 31 were assigned to factor 2 (negative reinforcement). For the evaluation of the questionnaire the Items 4,6,8,10,11,16,17,21,22,26,27,28 and 32 needed to be recoded. For example, a score of 7 in item 4 had to be recoded into a score of 1.

**Analysis of nicotine, cotinine, and hydroxycotinine plasma concentrations**

Multiple reaction monitoring (MRM) mode was used for mass selective detection in positive ionization mode with a 120s detection window and 1s cycle time. Two characteristic fragmentation reactions were used as transitions per analyte. Parameters for ESI source and scheduled MRM are given in Supplementary Tables 1 and 2.

**Supplementary Table 1.** Ion source parameters for nicotine, cotinine, and hydroxycotinine quantitation method.

| Ion spray voltage | 3800 V |
| --- | --- |
| Ion source temperature | 650 °C |
| Curtain gas | N_2_, 10 psi |
| Ion source gas 1 | N_2_, 80 psi |
| Ion source gas 2 | N_2_, 85 psi |
| Declustering potential | 47 V |
| Entrance potential | 7 V |

**Supplementary Table 2.** MRM parameters for nicotine, cotinine, and hydroxycotinine quantitation method.

| Analyte | Retention time | Quantifier | | | Qualifier | | |
| --- | --- | --- | --- | --- | --- | --- | --- |
|  |  | Q1 (Da) 🡪 Q3 (Da) | CE (V) | CXP (V) | Q1 (Da) 🡪 Q3 (Da) | CE (V) | CXP (V) |
| Nicotine | 3.2 min | 163.2 🡪 130.0 | 29 | 6 | 163.2 🡪 132.1 | 21 | 24 |
| Cotinine | 5.2 min | 177.2 🡪 98.0 | 40 | 18 | 177.2 🡪 80.0 | 25 | 14 |
| Hydroxycotinine | 4.4 min | 193.1 🡪 80.0 | 43 | 14 | 193.1 🡪 134.1 | 27 | 24 |
| Nicotine-d_3_ | 3.2 min | 166.3 🡪 132.0 | 23 | 6 | 166.3 🡪 130.0 | 45 | 6 |
| Cotinine-d_3_ | 5.2 min | 180.2 🡪 80.0 | 35 | 14 | 180.2 🡪 101.0 | 31 | 18 |
| Hydroxycotinine-d_3_ | 4.4 min | 196.2 🡪 80.0 | 41 | 14 | 196.2 🡪 134.1 | 27 | 24 |

CE: Collision energy
CXP: Collision exit potential

**Supplementary information on results**

**Plasma nicotine levels at different time points**

**Supplementary Table 3.** Results of plasma analysis for cigarette smokers. Excluded or missed values are highlighted in grey.

| Cigarette smoker | NMR | Plasma nicotine (ng/mL) | | | | | | | | | AUC_0-30_  ng/mL*min |
| --- | --- | --- | --- | --- | --- | --- | --- | --- | --- | --- | --- |
|  |  | t0 | 1 min | 2 min | 4 min | 6 min | 8 min | 10 min | 12 min | 30 min |  |
| 01 | 1.40 | 0.2 | 0.3 | 1.1 | 3.5 | 6.4 | **7.4** | 6.4 | 7.4 | 7.8 | 187.3 |
| 02 | 0.39 | 0.8 | 7.3 | 21.9 | 28.9 | **37.4** | 20.5 | 16.6 | 17.6 | 9.4 | 483.7 |
| 03 | 0.46 | 0.5 | 1.2 | 3.8 | 7.5 | **9.8** | N/A | N/A | 9.9 | 11.4 | 267.5 |
| 04 | 0.67 | 0.5 | 0.7 | 1.1 | 4.4 | 5.3 | 7.6 | 7.2 | **8.1** | 7.3 | 183.7 |
| 05 | 0.45 | 2.4 | 2.3 | 7.5 | 13.1 | **19.3** | N/A | N/A | 11.7 | 8.8 | 266.4 |
| 06 | 0.63 | 0.3 | 0.4 | 0.8 | 5.4 | 7.0 | 8.2 | **10.3** | N/A | 8.8 | 226.2 |
| 07 | 0.48 | 6.0 | 6.0 | 5.8 | 7.8 | 9.8 | 11.4 | **12.4** | 11.5 | 9.8 | 125.0 |
| 08 | nq | 0.0* | 0.0* | 0.0* | 0.0* | 0.1* | 0.1* | 0.1* | 0.1* | 0.0* | N/A |
| 09 | 0.58 | 0.1 | 0.4 | 2.3 | 5.6 | **8.6** | 7.9 | 6.5 | N/A | 6.0 | 178.4 |
| 10 | 0.29 | 5.5 | 10.3 | 14.8 | 21.8 | 25.5 | 24.2 | **26.3** | 25.9 | 14.5 | 454.4 |
| 11 | 0.62 | 0.2 | 0.6 | 7.8 | 21.5 | **26.6** | 21.3 | 9.7 | N/A | 4.5 | 297.4 |
| 12 | 0.61 | 0.1 | 0.2 | 0.7 | 3.4 | 6.8 | 10.0 | **10.8** | 9.2 | 5.4 | 201.0 |
| 13 | 0.25 | 0.3 | 0.9 | 2.4 | 6.9 | **7.8** | 7.7 | 6.4 | 6.4 | N/A | N/A |
| 14 | 0.16 | 0.6 | 1.0 | 3.1 | 10.9 | 17.1 | **17.1** | N/A | N/A | 11.3 | 374.5 |
| 15 | 0.28 | 0.0 | 0.5 | 7.1 | 18.3 | **29.1** | 18.0 | 15.3 | 14.4 | 3.9 | 351.5 |

NMR: Nicotine metabolic ratio (hydroxycotinin/cotinin concentration at t_0_)
AUC_0-30_: Area under the curve t_0_-t_30min_ (after subtraction of C_(t0)_)
N/A: not assessed (no blood sampling or no AUC_0-30_ calculation possible)
nq: not quantified (at least one metabolite concentration not quantifiable)
*: Not included in calculation of mean values

**Supplementary Table 4.** Results of plasma analysis for users of new/modified JUUL e-cigarettes. Excluded or missed values are highlighted in grey.

| Users new JUUL | NMR | Used liquid  (mg) | Nicotine dose (mg) | Plasma nicotine (ng/mL) | | | | | | | | | AUC_0-30_ ng/mL*min |
| --- | --- | --- | --- | --- | --- | --- | --- | --- | --- | --- | --- | --- | --- |
|  |  |  |  | t0 | 1 min | 2 min | 4 min | 6 min | 8 min | 10 min | 12 min | 30 min |  |
| 01 | 0.27 | N/A | N/A | 0.7 | 0.9 | 2.6 | 4.6 | 5.0 | **5.0** | N/A | 3.8 | 2.4 | 82.3 |
| 02 | 0.42 | 32.6 | 0.50 | 0.1 | 0.2 | 1.4 | 4.4 | 5.2 | **6.5** | 4.5 | N/A | 1.7 | 96.6 |
| 03 | 0.23 | 28.7 | 0.44 | 1.2 | 2.1 | 3.3 | **5.8** | 5.7 | 4.3 | 3.8 | 3.3 | 2.4 | 67.4 |
| 04 | 0.36 | 41.3 | 0.63 | 4.4 | 4.7 | 8.1 | 12.3 | **14.2** | 12.4 | 10.6 | 9.7 | 7.0 | 146.6 |
| 05 | 0.37 | 40.5 | 0.62 | 2.5 | 8.1 | 11.5 | 12.8 | **15.4** | 11.9 | N/A | N/A | 8.0 | 239.3 |
| 06^‡^ | 0.56 | 37.8 | 0.58 | 0.0 | 1.0 | 6.1 | **11.5** | 9.4 | 7.0 | 4.9 | 4.7 | 4.3 | 161.5 |
| *06•* | *0.55* | *28.9* | *0.44* | *0.9* | *1.1* | *3.9* | *N/A* | *N/A* | *N/A* | *4.2* | *4.1* | *3.4* | *N/A* |
| 07 | nq | 36.8 | 0.56 | 0.0 | 0.1 | 5.4 | 6.0 | **7.0** | 5.3 | N/A | N/A | 1.9 | 118.8 |
| 08^‡^ | 0.51 | 33.7 | 0.51 | 1.0 | 2.9 | 4.4 | 4.9 | **5.4** | 4.5 | 4.0 | 3.9 | 3.0 | 82.7 |
| *08•* | *0.93* | *32.9* | *0.50* | *0.3* | *1.6* | *4.5* | *4.2* | *N/A* | *N/A* | *2.3* | *3.2* | *1.6* | *N/A* |
| 09 | 0.39 | 32.2 | 0.49 | 0.4 | 1.5 | 2.7 | 5.0 | **6.9** | 4.6 | N/A | N/A | N/A | N/A |
| 10 | nq | 23.8 | 0.36 | 0.1 | 0.3 | 1.6 | 2.6 | **2.9** | N/A | 1.8 | 1.6 | 1.0 | 44.7 |
| 11 | 0.27 | 20.1 | 0.31 | 0.4 | 0.9 | N/A | 3.0 | N/A | N/A | N/A | N/A | N/A | N/A |
| 12 | 0.39 | 30.6 | 0.47 | 4.5 | 16.4 | N/A | N/A | N/A | N/A | N/A | N/A | N/A | N/A |
| 13^‡^ | 0.56 | 17.4 | 0.27 | 0.9 | 3.3 | 5.3 | **7.8** | 8.2 | 5.7 | N/A | 3.7 | N/A | N/A |
| *13•* | *0.42* | *21.5* | *0.33* | *4.3* | *2.2* | *9.7* | *N/A* | *N/A* | *N/A* | *N/A* | *N/A* | *3.8* | *N/A* |
| 14^‡^ | 0.16 | 46.0 | 0.70 | 0.0 | 1.2 | 6.6 | **11.2** | 9.9 | 7.0 | 5.5 | 4.6 | 3.6 | 156.6 |
| *14•* | *0.42* | *36.0* | *0.55* | *0.0* | *N/A* | *N/A* | *N/A* | *N/A* | *2.9* | *3.6* | *2.9* | *1.8* | *N/A* |
| 15 | 0.46 | 25.2 | 0.38 | 0.5 | 1.4 | **4.4** | 3.6 | 3.3 | N/A | N/A | N/A | 2.3 | 68.7 |

NMR: Nicotine metabolic ratio (hydroxycotinin/cotinin concentration at t_0_)
AUC_0-30_: Area under the curve t_0_-t_30min_ (after subtraction of C_(t0)_)
N/A: not assessed (no blood sampling or no AUC_0-30_ calculation possible)
nq: not quantified (at least one metabolite concentration not quantifiable)
‡: Second recruitment due to missing t_max_ sampling
•: Omitted (first) measurement due to missing t_max_ sampling

**Supplementary Table 5.** Results of plasma analysis for users of old/initial JUUL e-cigarettes. Excluded or missed values are highlighted in grey.

| Users old JUUL | NMR | Used liquid  (mg) | Nicotine dose (mg) | Plasma nicotine (ng/mL) | | | | | | | | | AUC_0-30_ ng/mL*min |
| --- | --- | --- | --- | --- | --- | --- | --- | --- | --- | --- | --- | --- | --- |
|  |  |  |  | t0 | 1 min | 2 min | 4 min | 6 min | 8 min | 10 min | 12 min | 30 min |  |
| 01 | 0.59 | 44.5 | 0.68 | 0.4 | 4.3 | **10.2** | 8.9 | 8.9 | 5.9 | 4.8 | 4.4 | 2.8 | 133.4 |
| 02 | 0.44 | 36.2 | 0.55 | 1.5 | 4.1 | 8.6 | **11.7** | 10.9 | 8.9 | N/A | 5.9 | 3.5 | 140.9 |
| 03 | 0.98 | 42.8 | 0.65 | 0.2 | 0.4 | 1.0 | **3.0** | 2.7 | 2.9 | 2.5 | 2.8 | 1.7 | 60.8 |
| 04 | 0.27 | 32.6 | 0.50 | 5.2 | 6.9 | 8.8 | 11.0 | **11.9** | 10.8 | 9.8 | 10.4 | 8.6 | 135.1 |
| 05 | 0.21 | 28.0 | 0.43 | 0.4 | 4.2 | **9.6** | 9.5 | 7.9 | 5.6 | 5.1 | N/A | 3.0 | 138.5 |
| 06 | 0.25 | 29.1 | 0.44 | 1.7 | 2.1 | 3.7 | **5.7** | 4.9 | 5.2 | **N/A** | 5.4 | 3.9 | 87.6 |
| 07 | 0.27 | 32.2 | 0.49 | 0.0 | 1.6 | 6.5 | **9.1** | 8.3 | 5.9 | 5.4 | 4.4 | 2.4 | 134.8 |
| 08 | 0.43 | 13.9 | 0.21 | 1.1 | 1.4 | 3.2 | **4.1** | 3.8 | 4.0 | 3.5 | 3.5 | N/A | N/A |
| 09 | 0.47 | 38.1 | 0.58 | 10.2 | 17.6 | **24.7** | 22.5 | 20.4 | 17.6 | 16.1 | 15.8 | 12.6 | 178.4 |
| 10 | 0.51 | 29.8 | 0.45 | 0.0 | 9.0 | **9.9** | 5.9 | 5.3 | 4.7 | 4.1 | N/A | 2.0 | 121.0 |
| 11 | 0.26 | 8.9 | 0.14 | 1.2 | 2.0 | 3.0 | **4.6** | 4.3 | 3.4 | N/A | N/A | 2.1 | 51.7 |

NMR: Nicotine metabolic ratio (hydroxycotinin/cotinin concentration at _t0_)
AUC_0-30_: Area under the curve t_0_-t_30min_ (after subtraction of C_(t0)_)
N/A: not assessed (no blood sampling, metabolite concentration not quantifiable or no AUC_0-30_ calculation possible)

**FTND and QSU-G scores for individual participants**

**Supplementary Table 6.** FTND and QSU-G scored for tobacco cigarette smokers. Excluded or missed values are highlighted in grey.

| Cigarette smoker | 01 | 02 | 03 | 04 | 05 | 06 | 07 | 08 | 09 | 10 | 11 | 12 | 13 | 14 | 15 |
| --- | --- | --- | --- | --- | --- | --- | --- | --- | --- | --- | --- | --- | --- | --- | --- |
| FTND | 1 | 2 | 4 | 0 | 4 | 1 | 0 | 0* | 0 | 7 | 0 | 0 | 1 | 0 | 1 |
| QSU-G Factor 1 before | 4.27 | 6.27 | 5.82 | 4.00 | 6.00 | 4.00 | 3.64 | N/A | 4.64 | 6.55 | 4.45 | 2.64 | 4.73 | 4.00 | 3.91 |
| QSU-G Factor 1 after | 4.27 | 5.18 | 6.09 | 2.18 | 3.82 | 4.09 | 3.82 | N/A | 5.09 | 4.55 | 2.64 | 2.45 | 3.82 | 3.09 | 2.18 |
| QSU-G Factor 2 before | 2.40 | 3.40 | 2.30 | 1.40 | 3.90 | 1.70 | 2.20 | N/A | 2.50 | 1.60 | 3.50 | 2.10 | 2.50 | 1.00 | 2.60 |
| QSU-G Factor 2 after | 2.60 | 2.30 | 2.90 | 1.10 | 2.60 | 1.80 | 2.10 | N/A | 3.10 | 1.40 | 2.20 | 2.00 | 2.00 | 1.00 | 1.90 |

*: Not included in calculation of mean values
N/A: no QSU-G score calculation due to prior exclusion

**Supplementary Table 7.** FTND and QSU-G scored for users of new/modified JUUL. Excluded or missed values are highlighted in grey.

| New JUUL user | 01 | 02 | 03 | 04 | 05 | 06 | 07 | 08 | 09 | 10 | 11 | 12 | 13 | 14 | 15 |
| --- | --- | --- | --- | --- | --- | --- | --- | --- | --- | --- | --- | --- | --- | --- | --- |
| FTND | 2 | 0 | 8 | 5 | 5 | 3 | 0 | 2 | 1 | 2 | 7 | 8 | 2 | 3 | 8 |
| QSU-G Factor 1 before | 3.36 | 3.64 | 6.27 | 4.00 | 6.00 | 6.45 | 3.64 | 2.73 | 1.09 | 5.18 | 6.73 | N/A | 5.36 | 3.00 | 7.00 |
| QSU-G Factor 1 after | 4.64 | 5.09 | 6.91 | 1.73 | 4.55 | 5.55 | 2.91 | 3.09 | 3.91 | 4.00 | 5.55 | N/A | 3.73 | 3.27 | 7.00 |
| QSU-G Factor 2 before | 1.30 | 1.40 | 4.80 | 1.50 | 2.10 | 4.80 | 1.60 | 1.70 | 1.00 | 2.40 | 2.40 | N/A | 2.80 | 1.30 | 4.10 |
| QSU-G Factor2 after | 1.10 | 1.30 | 4.90 | 1.00 | 2.50 | 2.50 | 1.60 | 2.20 | 1.00 | 1.60 | 2.80 | N/A | 1.20 | 1.80 | 5.50 |

N/A: no score calculation due to incomplete participation in questionnaire

**Supplementary Table 8.** FTND and QSU-G scored for users of old/initial JUUL. Excluded or missed values are highlighted in grey.

| Old JUUL user | 01 | 02 | 03 | 04 | 05 | 06 | 07 | 08 | 09 | 10 | 11 |
| --- | --- | --- | --- | --- | --- | --- | --- | --- | --- | --- | --- |
| FTND | 0 | 5 | 2 | 5 | 2 | 8 | 0 | 5 | 8 | 3 | N/A |
| QSU-G Factor 1 before | 4.09 | 4.91 | 2.27 | 3.55 | N/A | 6.82 | 3.45 | 4.82 | 6.27 | 3.27 | N/A |
| QSU-G Factor 1 after | 5.18 | 4.36 | 3.45 | 2.82 | N/A | 6.82 | 2.82 | 5.27 | 6.64 | 3.64 | N/A |
| QSU-G Factor 2 before | 1.20 | 3.10 | 1.80 | 1.10 | N/A | 5.20 | 1.80 | 1.80 | 2.30 | 1.90 | N/A |
| QSU-G Factor2 after | 1.80 | 2.80 | 2.40 | 1.00 | N/A | 4.50 | 1.50 | 1.80 | 2.90 | 2.30 | N/A |

N/A: no score calculation due to incomplete participation in questionnaire

**Cotinine and hydroxycotinine plasma concentration-time curves**

Ratios of the plasma concentrations of metabolites hydroxycotinine at t_0_ were calculated as a surrogate for nicotine metabolism. However, metabolites were determined at the other time points as well. Plasma concentration-time curves per group and analyte are presented in Supplementary Figure 1 for sake of completeness.


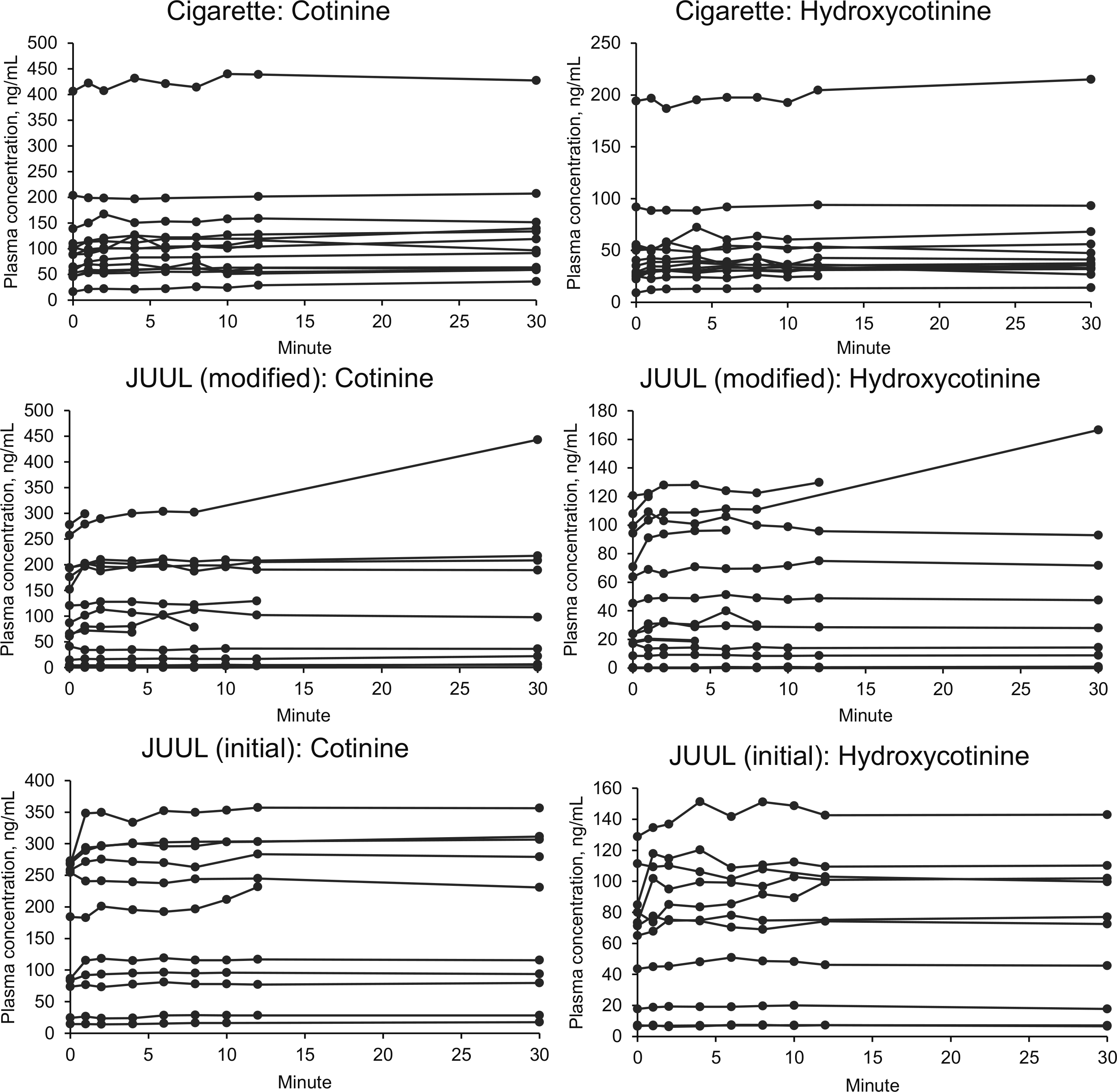


**Supplementary Figure 1.** Individual plasma concentration-time curves for metabolites cotinine and hydroxycotinine derived from the three study groups.

**References**

1 Müller, V., Mucha, R. F., Ackermann, K. & Pauli, P. Die Erfassung des Cravings bei Rauchern mit einer deutschen Version des “Questionnaire on Smoking Urges“ (QSU-G). *Zeitschrift für Klinische Psychologie und Psychotherapie*. **30**, 164-71 (2001).
